# Texture-Based Preprocessing Framework with nnU-Net Model for Accurate Intracranial Artery Segmentation

**DOI:** 10.3390/jimaging11120438

**Published:** 2025-12-09

**Authors:** Kyuseok Kim, Ji-Youn Kim

**Affiliations:** 1 Institute of Human Convergence Health Science, Gachon University, 191, Hambakmoero, Yeonsu-gu, Incheon 21936, Republic of Korea; kskim502@gachon.ac.kr; 2 Department of Dental Hygiene, Gachon University, 191, Hambakmoero, Yeonsu-gu, Incheon 21936, Republic of Korea

**Keywords:** cerebrovascular extraction, texture analysis, preprocessing, deep learning, time sequence, digital subtraction angiography

## Abstract

Accurate intracranial artery segmentation from digital subtraction angiography (DSA) is critical for neurovascular diagnosis and intervention planning. Vascular extraction, which combines preprocessing methods and deep learning models, yields a high level of results, but limited preprocessing results constrain the improvement of results. We propose a texture-based contrast enhancement preprocessing framework integrated with the nnU-Net model to improve vessel segmentation in time-sequential DSA images. The method generates a combined feature mask by fusing local contrast, local entropy, and brightness threshold maps, which is then used as input for deep learning–based segmentation. Segmentation performance was evaluated using the DIAS dataset with various standard quantitative metrics. The proposed preprocessing significantly improved segmentation across all metrics compared to both the baseline and contrast-limited adaptive histogram equalization (CLAHE). Using nnU-Net, the method achieved a Dice Similarity Coefficient (DICE) of 0.83 ± 0.20 and an Intersection over Union (IoU) of 0.72 ± 0.14, outperforming CLAHE (DICE 0.79 ± 0.41, IoU 0.70 ± 0.23) and the baseline (DICE 0.65 ± 0.15, IoU 0.47 ± 0.20). Most notably, vessel connectivity (VC) dropped by over 65% relative to unprocessed images, indicating marked improvements in VC and topological accuracy. This study demonstrates that combining texture-based preprocessing with nnU-Net delivers robust, noise-tolerant, and clinically interpretable segmentation of intracranial arteries from DSA.

## 1. Introduction

Cerebrovascular diseases (e.g., stroke, aneurysm) are a leading cause of death and disability worldwide, with incidence doubling every decade after age 55 [1]. The timely and precise diagnosis of cerebrovascular diseases is imperative, as any delay in treatment can result in permanent neurological impairments; therefore, an expeditious imaging assessment is essential. Digital Subtraction Angiography (DSA) remains the gold standard imaging technique for diagnosing intracranial vascular conditions, providing high-resolution, real-time visualization of cerebral vessels and blood flow [2,3]. However, interpreting DSA sequences relies on labor-intensive, frame-by-frame analysis by experts, which is time-consuming and prone to observer variability. Small vessels and overlapping structures make manual delineation challenging [4]. Consequently, the development of automated cerebrovascular segmentation techniques is a high priority. Such systems support clinicians by providing an objective visualization of vascular structures and pathologies, thereby facilitating more rapid diagnostics and enhancing preoperative planning.

Traditional image-processing approaches have been widely used to extract blood vessels from DSA images. A common technique is thresholding, which subtracts background by binarizing the image, this can successfully isolate major vessels under ideal conditions [5]. Other methods enhance vessel visibility through filters. For example, contrast-limited adaptive histogram equalization (CLAHE) and the Frangi vesselness filter emphasize the tubular structure of vessels to separate them from background tissue [6]. Graph-based approaches (e.g., marker-controlled watershed or minimum-cost graph cuts) use connectivity and edge information to delineate vessel networks [7,8]. These classical techniques, represent the established practice for vessel extraction in many settings. Despite demonstrating some success, these methods remain highly sensitive to noise and variability. They frequently struggle to adapt to varying background intensities, often resulting in the omission of distal blood vessels or the inclusion of non-vascular structures. In practice, these often leave unwanted anatomy (bone edges, catheters) in the result, requiring manual removal that is time-consuming and non-standardized between operators.

Recent advances in artificial intelligence have significantly improved diagnostic imaging workflows across various specialties, including neurology, cardiovascular medicine, and interventional radiology [9]. Convolutional neural networks (CNNs), particularly the U-Net encoder–decoder architecture, have demonstrated outstanding performance in medical image segmentation and have quickly become foundational in vessel extraction tasks. Early applications of U-Net and its variants focused on individual DSA frames. For instance, Zhang et al. employed a deep U-shaped network to segment intracranial arteries in single-frame DSA images, thereby validating the feasibility of data-driven cerebrovascular extraction [10]. Subsequent advancements introduced enhanced models, such as multi-scale CNN, which integrated multi-resolution contextual features and offered moderate performance gains over the conventional U-Net architecture [11]. Further improvements were achieved through the development of specialized architectures, including multiscale dense networks and edge-regularized models, specifically tailored to detect fine vascular structures and suppress spurious boundaries [12]. While these deep learning-based methods surpassed traditional segmentation techniques in delineating major cerebral vessels, their reliance on single-frame input limited their effectiveness. Specifically, they struggled with challenges intrinsic to DSA imaging, such as poor visualization of small-caliber vessels, misinterpretation of static anatomical backgrounds, and fragmented vessel representations when complete filling occurred only in alternate frames. To overcome these issues, recent techniques leverage the temporal dimension of DSA sequences. Rather than analyzing one image at a time, sequence-based models process the entire time series to capture the dynamic flow of contrast through the vasculature. For instance, the CAVE model introduced a spatio-temporal U-Net that integrates convolutional encoders with recurrent units, allowing it to learn both spatial vessel features and their evolution over time. Such spatio-temporal networks can accumulate information across frames, revealing vessels that appear faint or momentarily in individual images [13]. Wang et al. developed DSANet, which uses parallel temporal and spatial encoders to fuse motion cues from the sequence, achieving state-of-the-art accuracy on DSA segmentation tasks [14]. These methods significantly outperform single-frame models and even classical vessel filters, because incorporating contrast flow continuity yields more complete and robust vessel maps. Deep learning has thus pushed cerebrovascular extraction forward, enabling detection of smaller and more complex vessel structures than previously possible. Despite their success, deep learning-based techniques have limitations that ongoing research is working to address. A key challenge is the need for large, high-quality annotated datasets to train these models. DSA sequence data with expert labels have been scarce due to the invasive nature of angiography and the effort required for manual annotation. Until recently, the lack of public DSA datasets impeded progress, although new resources like the DIAS and DSCA sequence datasets have started to fill this gap [15,16]. Data scarcity has also spurred interest in weakly supervised learning. For instance, methods that learn from partial annotations or generate pseudo-labels using scribbles or active contour models to reduce the burden of full pixel-wise labeling.

Preprocessing the DSA images is critically important for successful deep learning-based vessel segmentation. Effective preprocessing enhances vessel contrast while suppressing background noise, thus providing the neural network with a clearer image to learn from. Previously, based on CLAHE and Frangi filtering, increasing the contrast between blood vessels and background or performing initial vascular extraction and then using them as inputs for deep learning models are typical [17,18]. These approaches excel at making tubular features more prominent and improving image contrast. However, when used on their own, they can also intensify noise and create misleading results by enhancing random patterns as if they were vessels. Since raw DSA images contain substantial noise, a vital preprocessing step is required to boost vessel-to-background contrast without also magnifying these unwanted artifacts. Another example is advanced filtering algorithms designed for angiographic data: Qin et al. introduced a tensor completion method to decompose X-ray angiograms into a low-rank background layer and a sparse vessel layer [19]. By removing the background through tensor decomposition, the vessels are isolated in a “cleaner” image, which can then be segmented with fewer false alarms from bony structures or noise. However, tensor complementation-based decomposition separates the background and blood vessels based on the low-rank + sparse structure assumption. However, it has also been mentioned in experiments that sparse and thin blood vessels (especially peripheral blood vessels) are not sufficiently reflected as a sparse term or can be considered as noise in the thresholding process and lost. In addition, the proposed tensor complementation method includes complex matrix optimization algorithms such as t-SVD, nuclear norm, and L1-norm, so the computational cost is high to use as a preprocessing step. In addition, as specified in the paper, several threshold and hyperparameter settings are sensitive, requiring optimization adjustment according to training data or resolution. Researchers are creating specialized preprocessing techniques for angiography, a key example being the integration of learnable modules into the segmentation workflow. For instance, Iyer et al. introduced an Angiographic Processing Network (APN) that serves as a front-end to a segmentation model [20]. This APN is trained to find the optimal way to filter and normalize images before they are segmented. By training the APN jointly with a DeepLabv3+ segmenter, the system learned to automatically apply the most suitable enhancements, such as contrast stretching or denoising, to every frame. This method resulted in significantly better vessel segmentation compared to using conventional fixed filters, demonstrating that improving the input data representation is just as crucial for boosting accuracy as refining the segmentation model itself. However, when APN learns a global contrast enhancement strategy, it is likely that relatively thin and faint structures will not be emphasized.

In addition to methods that analyze pixel intensity, texture analysis techniques are also employed to assist in cerebrovascular segmentation. This approach is effective because blood vessels exhibit distinct textural characteristics, appearing as long, winding structures that stand out against a background of smoother tissues and noise. Combined with morphological preprocessing and contrast enhancement, yielded high accuracy in segmenting retinal blood vessels by capturing their texture signatures [21,22]. Recent advances in image preprocessing have demonstrated that integrating localized image statistics, such as local contrast and local entropy, significantly enhances the accuracy of vessel segmentation models. A vessel extraction framework combining local phase-preserving denoising with local normalization and maximum entropy thresholding achieved superior performance on retinal datasets by accounting for regional texture variations and suppressing background interference [23]. Similarly, Yin et al. applied Hessian-based line enhancement in conjunction with localized entropy binarization to capture fine vascular structures, demonstrating that vessel presence correlates strongly with localized entropy patterns and contrast gradients [24]. These studies highlight the efficacy of spatially adaptive thresholds in biomedical imaging domains where structural complexity, illumination variance, and background clutter pose significant challenges. Local entropy functions as a texture-sensitive criterion, emphasizing regions with high signal variation, while local contrast normalizes uneven illumination and enhances edge transitions. Brightness thresholding further distinguishes hyperintense vascular signals from adjacent anatomical noise, especially in contrast-enhanced angiographic sequences.

This study aims to improve the cerebrovascular extraction performance of a deep learning model that modifies the texture analysis techniques in time series DSA data to perform preprocessing and uses it as an input data.

## 2. Materials and Methods

### 2.1. Dataset

This study utilized the Digital Subtraction Angiography Intracranial Artery Segmentation dataset (DIAS) [15], a publicly available benchmark dataset specifically designed for evaluating segmentation performance on time-sequential DSA images of intracranial arteries. The DIAS dataset was curated and released to support research in cerebrovascular image analysis and automated vessel segmentation.

All DSA sequences were collected at Beijing Tiantan Hospital during routine neuro-interventional procedures performed between January 2019 and December 2021. The dataset was retrospectively constructed under ethical approval and fully anonymized prior to release. According to the original data release, the sequences were obtained with standard clinical protocols using biplane angiographic systems. From the source pool of over 1000 clinical DSA sequences, 120 sequences were selected for this study based on specific inclusion criteria: Clear visualization of the arterial phase; Minimal motion artifacts; and Absence of sequence duplication.

These selected sequences contain a total of 762 arterial-phase frames, with each sequence consisting of 4 to 14 frames, acquired at a frame rate of 4 frames per second (fps). All images are provided at a spatial resolution of 800 × 800 pixels, and are available in either anteroposterior or lateral views. The patient cohort primarily includes cases diagnosed with intracranial atherosclerotic stenosis and middle cerebral artery occlusion, providing a representative sample of cerebrovascular pathologies.

To ensure high annotation fidelity, each DSA frame was labeled following a semi-automatic annotation protocol. Initially, two medical students conducted manual annotations of key frames from the early and late arterial phases. These annotations were then reviewed and verified by two board-certified neurosurgeons. Label propagation was applied to extend annotations across intermediate frames within each sequence, balancing annotation quality and efficiency. The final dataset includes binary vessel masks for all frames, serving as the ground truth for training and evaluation.

This retrospective study was approved by the Institutional Review Board (IRB) of Gachon University (Approval No. 1044396-202503-HR-051-01), which waived the requirement for informed consent due to the use of a publicly available, anonymized dataset. The IRB review waiver was granted on 2 April 2025. All experiments were conducted using the DIAS dataset with a sequence-level split to prevent frame-level data leakage. The dataset was divided into 70% for training, 10% for validation, and 20% for testing, ensuring that no frames from the same DSA sequence appeared in more than one subset.

### 2.2. Proposed Method

**Figure 1:**
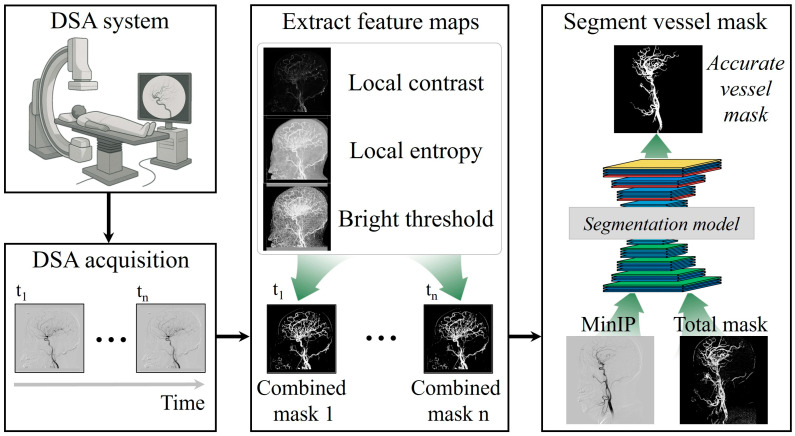
Simplified framework to extract texture-based feature maps from time-series digital subtraction angiography (DSA) images and combine them for deep learning-based segmentation to generate accurate cerebral vessel masks.

Figure 1 shows the proposed scheme to segment the cerebral artery brain vessel of projection domain in DSA. Briefly, the proposed framework begins with the acquisition of time-sequential DSA images using a conventional angiography system. The image sizes were 800 × 800. Multiple frames are captured over time to document the passage of contrast material through the cerebral vasculature. These temporal DSA sequences form the foundation of the method, providing both structural and dynamic vascular information that can be leveraged for enhanced vessel delineation. By systematically processing frames, the approach ensures that subtle vascular features are preserved across the entire acquisition period. In the second stage, the acquired DSA frames undergo texture-based feature extraction to generate enhanced vessel representations. Three complementary texture descriptors including local contrast, local entropy, and brightness thresholding, are applied to each frame, isolating vessels from background structures and improving the visibility of small-caliber branches [25,26,27].

Local contrast refers to the measurement of intensity variation within a defined neighborhood around each pixel as Equation (1):

(1) $$C(x,y)=\sqrt{\frac{1}{N}\sum_{i,j\in N(x,y)}{\left(I\left(i,j\right)-\mu (x,y)\right)}^{2}}$$

where $I\left(i,j\right)$ is intensity of pixel, μ(x,y) is average brightness of neighbor N(x,y) based on center (x,y), and N is number of pixels in window. It plays a critical role in enhancing fine structural details in medical imaging. Unlike global contrast adjustments, which modify the overall brightness and contrast of an entire image, local contrast selectively amplifies differences in pixel intensity at a regional level, making subtle features such as thin or low-contrast vessels more distinguishable from surrounding tissues. This is particularly important for digital subtraction angiography, where small-caliber vessels may be obscured by overlapping bone structures or background noise in the raw projection images. In the proposed framework, local contrast is computed by analyzing the difference between a target pixel’s intensity and the average or weighted intensity of its local neighborhood. A common approach involves a sliding window that calculates local statistics, such as mean and standard deviation, and highlights regions where the target pixel deviates significantly from its surroundings. In this study, we used 5 × 5 pixels, empirically. This process enhances edges and vessel boundaries by increasing the relative visibility of regions with abrupt intensity changes, while suppressing uniform areas that contribute little to structural information. The advantage of using local contrast in cerebrovascular segmentation lies in its ability to preserve fine vascular structures without globally over-amplifying noise. When combined with other texture descriptors like local entropy and brightness thresholding, local contrast contributes to a more balanced enhancement process: it emphasizes the spatial definition of vessels, ensures that low-contrast branches are not lost, and provides the segmentation network with richer, vessel-focused features. This targeted enhancement helps the model delineate vessel edges more accurately and maintain continuity even in areas where the vessel signal is faint or partially obscured.

Local entropy is a statistical texture descriptor that quantifies the degree of randomness or complexity in the pixel intensity distribution within a defined local neighborhood. Unlike global entropy, which measures overall image disorder, local entropy captures regional variations in information content, making it particularly effective for detecting areas with intricate structures, such as branching vessels in medical images. In DSA, regions with high vascular complexity typically exhibit higher entropy values because of the heterogeneous pixel intensities produced by overlapping small-caliber vessels, while homogeneous regions like soft tissue or uniform background show lower entropy. In the proposed framework, local entropy is computed by sliding a window (e.g., 5 × 5 pixels) across each DSA frame and calculating the Shannon entropy within that neighborhood. Shannon entropy is defined as:

(2) $$H\left(x,y\right)=-\sum_{k=0}^{L-1}p_{k}(x,y)\log_{2}\left(p_{k}\left(x,y\right)+\epsilon \right)$$

where $p_{k}(x,y)$ represents the probability, k, of each intensity level within the local window. L is number of gray-scale levels, and ϵ is to prevent logarithmic zero and returns a value of 2^−52^, the distance from 1.0 to the next largest allocation density number. High entropy values indicate a diverse range of intensities whereas low entropy values correspond to uniform regions lacking meaningful structural details. This calculation produces an entropy map that highlights areas rich in texture information and suppresses regions of little diagnostic value. The inclusion of local entropy in the preprocessing stage has two key benefits for cerebrovascular segmentation. First, it enhances small and branching vessels by emphasizing regions with complex intensity patterns, enabling the segmentation network to recognize and preserve finer anatomical details that might otherwise be overlooked. Second, by down-weighting homogeneous regions, local entropy helps reduce the influence of non-informative background, such as uniform soft tissue or flat bone shadows, which can confuse the segmentation model. When integrated with complementary descriptors like local contrast and brightness thresholding, local entropy provides an additional layer of contextual richness, ultimately leading to more accurate and continuous vessel extraction in deep learning based segmentation.

Bright thresholding is a technique in medical image processing that emphasizes or extracts pixels with intensities above a defined threshold, making it particularly useful for isolating structures such as blood vessels that appear relatively brighter than surrounding tissues. Vessels filled with contrast medium typically exhibit higher intensity values compared to background tissues or bone, meaning that by applying an appropriate threshold, nonvascular structures can be suppressed while vascular signals are selectively highlighted as Equation (3):

(3) $$T=Q\left(I\right)=\inf\left\{t\in R\right|P(I\leq t)\geq 0.85\}$$

where P(I≤t) is cumulative distribution function (CDF), and T is boundary value dividing the area brighter than the top 15% of all pixels. In this study, the bright threshold was applied not simply as a global cutoff but rather adapted to the characteristics of each frame or region. In this study, region refers to the entire frame, and the brightness threshold was computed independently for each DSA frame using its cumulative histogram. No sub-regional thresholding was applied. The histogram of each image was analyzed to determine the upper range of intensity values corresponding to vascular pixels, and those exceeding the threshold were identified as vessel candidates. This approach effectively removes uniformly bright structures like bones or device artifacts and eliminates darker, low-information regions, leaving only vessel-relevant signals. In frames where contrast distribution is uneven or noise is present, adaptive or locally computed thresholds can be employed to maintain signal fidelity and avoid excessive information loss. Window size, entropy threshold, and brightness percentile were tuned empirically on the validation set. Among the candidate settings (3 × 3, 5 × 5, 7 × 7 windows; 80th–90th percentiles), the combination of a 5 × 5 window and the 85th percentile provided optimal vessel-to-background separation while minimizing noise amplification.

**Figure 2:**
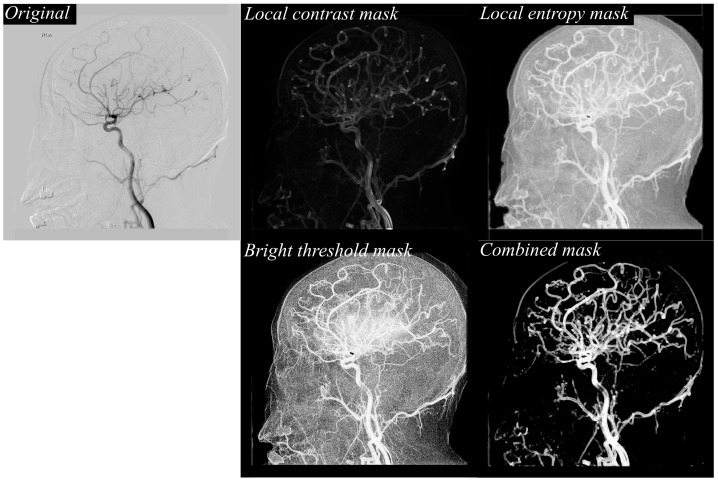
Example of texture-based feature extraction from a DSA image. The original DSA frame is processed to generate local contrast, local entropy, and bright threshold masks, which are then fused to form a combined mask emphasizing vascular structures for improved segmentation input.

These feature maps are then fused into combined masks for each time point, effectively consolidating multiple textural cues into a single, vessel-focused representation. This fusion step enhances vascular continuity while suppressing noise and irrelevant background details, thereby providing the segmentation model with clearer and more informative inputs. Figure 2 illustrates the stepwise texture-based feature extraction process applied to a representative DSA frame. The original image (top left) depicts the raw arterial-phase DSA projection, which contains overlapping vascular structures as well as background tissues and noise. While the primary vessels are visible, the image includes low-contrast regions and subtle branches that could be easily missed during direct segmentation, underscoring the need for enhanced preprocessing to clarify vessel structures before deep learning analysis. To address this, three complementary texture masks are generated from the original frame: the local contrast mask (top right) enhances boundaries and highlights regional intensity differences, allowing fine edges of vessels to become more distinct; the local entropy mask (middle right) identifies regions of high information content, emphasizing the complex, branching vascular areas while suppressing homogeneous regions such as flat bone shadows; and the bright threshold mask (bottom left) selectively isolates pixels above a defined intensity level, focusing on bright vessel signals and filtering out background interference. These masks are then fused into a single combined mask (bottom right), which consolidates the strengths of each texture descriptor. The combined mask retains fine peripheral branches, reinforces vessel continuity, and reduces noise, resulting in a vessel-focused representation optimized for deep learning segmentation. This fusion process not only improves the visibility of clinically important small-caliber vessels but also provides the segmentation model with a clean, enhanced input that supports more accurate and topologically coherent cerebrovascular extraction.

The proposed preprocessing method and a contrast-limited adaptive histogram equalization (CLAHE) method [28], which are typically used, were used for comparison verification. It can be defined as Equation (4).

(4) $$CLAHE\left(i\right)=\frac{cdf\left(i\right)-{cdf}_{min}}{\left(w_{x}\times w_{y}\right)-{cdf}_{min}}\times \left(G-1\right)$$

where i represents the pixel intensity of the DSA image. The input image is segmented into small, non-overlapping contextual regions or tiles (e.g., blocks of size $w_{x}$ by $w_{y}$). For each tile, a histogram of pixel intensities is generated and then converted into the CDF. G denotes the total number of gray levels present in the DSA images. To avoid excessive noise amplification in homogeneous regions, the histogram is clipped at a predefined threshold, and the excess counts are uniformly redistributed across the histogram. ${cdf}_{min}$ refers to the minimum CDF value within the tiles.

Finally, a series of combined masks is entered into the nnU-Net segmentation model [29]. As for the form of the input image, two total masks are entered by summing the feature maps of a minimum intensity projection (MinIP) [30] and the combined mask for each sequence. Dataset was divided into training, validation, and test at a ratio of 70:10:20, and the input data for deep learning was divided and inputted after sliding by 20 pixels with 256 × 256 pixels. The nnU-Net model was implemented as a 4-layer encoder–decoder architecture and approximately 9 million trainable parameters. Following the self-configuring principles of nnU-Net, the model automatically adapted its kernel sizes, feature map scaling, and normalization strategies to the dataset, while retaining the foundational U-Net design. In the encoder path, feature extraction was performed through two sequential 3 × 3 convolutional layers at each level, each followed by ReLU activation [31] and batch normalization [32], enabling robust feature representation even with varying image intensity distributions. Spatial downsampling was achieved using 2 × 2 max-pooling with a stride of two, progressively reducing the spatial resolution while increasing feature abstraction. The number of feature maps started at 32 in the first layer and doubled at each subsequent depth level to capture increasingly complex semantic representations. The decoder path symmetrically mirrored the encoder structure, employing 2 × 2 transposed convolutions for upsampling to restore spatial resolution, followed by two 3 × 3 convolutions with ReLU and instance normalization. Skip connections between encoder and decoder layers preserved fine-grained spatial information by concatenating corresponding feature maps, facilitating accurate localization of vessel structures. The number of feature maps was halved at each decoder level, returning to 32 channels before the final layer. Finally, a 1 × 1 convolution produced the binary segmentation map output, generating the vessel masks used for further processing in the framework. Five-fold data augmentation indicates that five augmentation operators including random rotation, horizontal flip, vertical flip, contrast jittering, and Gaussian noise injection were applied with 0.5 probability each during training. The deep learning model was trained using the Adam optimizer [33] with categorical cross-entropy as the loss function [34], and the initial learning rate was set to 1 × 10^−5^. Training was conducted for 50 epochs with a batch size of 50.

The computational environment consisted of a Windows 10 workstation with a 2.13 GHz CPU, 128 GB RAM, and an NVIDIA RTX 3090 GPU with 24 GB of memory. All image processing and model implementation were carried out using MATLAB (R2023a, MathWorks, Natick, MA, USA) and PyTorch (v2.6.0, Meta AI, New York, NY, USA).

### 2.3. Quantitative Metrics

Eight quantitative metrics were employed to comprehensively evaluate the performance of the proposed vascular extraction method: Dice Similarity Coefficient (DICE), Intersection over Union (IoU), Accuracy, Sensitivity, Specificity, Precision, F1-score, and Vessel Connectivity (VC) [35,36,37,38]. These metrics were selected to assess not only the pixel-wise agreement between the predicted and ground-truth masks but also the topological integrity of vascular structures.

The DICE quantifies the degree of overlap between the predicted vascular segmentation and the ground truth mask, and is defined as:

(5) $$DICE=\frac{2TP}{2TP+FP+FN}$$

where TP (True Positive), FP (False Positive), and FN (False Negative) represent correctly identified vessel pixels, incorrectly segmented vessel pixels, and missed vessel pixels, respectively. DICE values range from 0 to 1, with 1 indicating perfect agreement. IoU, also known as the Jaccard index, provides a stricter assessment by measuring the ratio of the intersection to the union between the predicted and ground truth masks:

(6) $$IoU=\frac{TP}{TP+FP+FN}$$

while similar to DICE and IoU penalizes mismatches more heavily, making it a more stringent performance metric.

Accuracy measures the proportion of correctly classified pixels (both vessel and background) across the entire image:

(7) $$Accuracy=\frac{TP+TN}{TP+TN+FP+FN}$$

However, because background pixels are often dominant, Accuracy alone is insufficient for imbalanced datasets. Sensitivity captures the proportion of actual vessel pixels correctly identified by the model:

(8) $$Sensitivity=\frac{TP}{TP+FN}$$

High Sensitivity ensures that subtle and thin vessel segments are not overlooked. Specificity reflects the proportion of correctly identified non-vessel pixels, indicating the model’s ability to avoid false vessel predictions:

(9) $$Specificity=\frac{TN}{TN+FP}$$

Precision evaluates the reliability of vessel predictions by quantifying how many of the pixels labeled as vessels are truly vessels:

(10) $$Precision=\frac{TP}{TP+FP}$$

F1-score is the harmonic mean of Precision and Sensitivity, providing a balanced performance indicator in datasets with class imbalance:

(11) $$F1-score=\frac{2\times Precision\times Sensitivity}{Precision+Sensitivity}$$

Pixel-based metrics do not capture vascular topology or continuity. Therefore, VC was included to assess whether the predicted vessel map preserves the branching patterns and uninterrupted paths of vascular structures. VC was calculated by comparing predicted and ground-truth vessel centerlines on a segment-wise basis:

(12) $$VC=\frac{1}{S}\sum_{p=1}^{S}CE(C_{p},D_{p})$$

where S is the number of vessel segments, $C_{p}$ and $D_{p}$ represent the ground truth and predicted centerlines of the p-th vessel segment, and CE(⋅) denotes the connectivity error derived from geodesic distances. To compute VC in practice, both the ground-truth and predicted vessel masks are first skeletonized using a thinning algorithm to obtain 1-pixel-wide centerlines. These centerlines are then decomposed into individual vessel segments, where each segment is defined as a continuous path between two branching points or between a branch point and a terminal endpoint. For each corresponding vessel segment in the ground truth, we compute the geodesic distance between the predicted and true centerlines. Geodesic distance is defined as the shortest path restricted to the vessel manifold, rather than a simple Euclidean distance, and is obtained by computing the minimal accumulated cost along the centerline graph. The connectivity error for a segment is defined as the mean geodesic distance between corresponding points on the two centerlines. If the predicted segment is fragmented or disconnected, additional penalty terms are added by summing the geodesic distances of all unmatched sub-segments, producing larger VC values. Lower VC values indicate better preservation of vascular continuity. VC offers a critical complement to conventional segmentation metrics because high DICE or IoU scores can sometimes mask clinically important errors. For instance, small peripheral vessels may be omitted without substantially lowering these overlap-based indices. In contrast, VC directly assesses the continuity and integrity of the vascular tree, assigning penalties when segmented vessels are fragmented, truncated, or disconnected from the main network. This makes VC especially valuable in evaluating whether an algorithm preserves fine vascular branches and ensures anatomically coherent vessel structures.

All statistical analyses were performed using SPSS Statistics software (version 21.0; SPSS Inc., Chicago, IL, USA). For each arterial brain vessel projection, all quantitative metrics—including DICE, IoU, Sensitivity, Specificity, Precision, F1-score, and Vessel Connectivity (VC)—were reported as mean values with corresponding standard deviations and 95% confidence intervals (CI). Statistical significance between preprocessing methods was assessed using the Wilcoxon paired non-parametric test because the performance metrics did not follow a normal distribution. A *p*-value < 0.05 was considered statistically significant.

## 3. Results

### 3.1. Evaluation of Visual Performance

**Figure 3:**
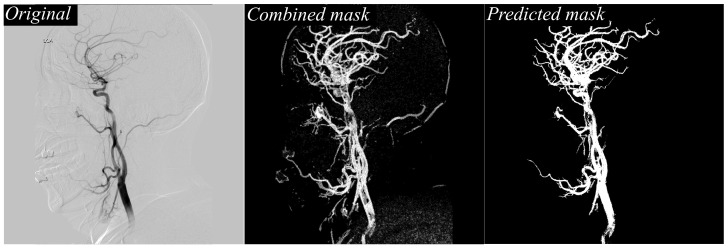
Comparison of original DSA image, texture-enhanced combined mask, and the final predicted vessel mask.

Figure 3 illustrates, in detail, the stepwise vessel segmentation process using the proposed framework. The figure begins with the original DSA image, which serves as the raw input from the angiographic system. Adjacent to it is the texture-enhanced combined mask, generated by integrating three complementary texture descriptors: local contrast, which highlights subtle intensity differences between vessel and background; local entropy, which captures the structural complexity and suppresses low-information regions; and the bright threshold map, which further isolates high-intensity vessel regions. By fusing these feature maps, the combined mask offers a highly refined representation of vascular structures, minimizing background noise while preserving fine vessel details. This enhanced mask is then fed directly into the deep learning segmentation model, resulting in the final predicted vessel mask. The predicted mask demonstrates sharp delineation of both major cerebral arteries and delicate peripheral branches, reflecting the model’s ability to faithfully leverage the enhanced texture information for improved segmentation performance. Importantly, even the small-caliber vessels maintain continuity, suggesting the framework effectively prevents the common issue of fragmented predictions often observed in conventional DSA segmentation workflows.

**Figure 4:**
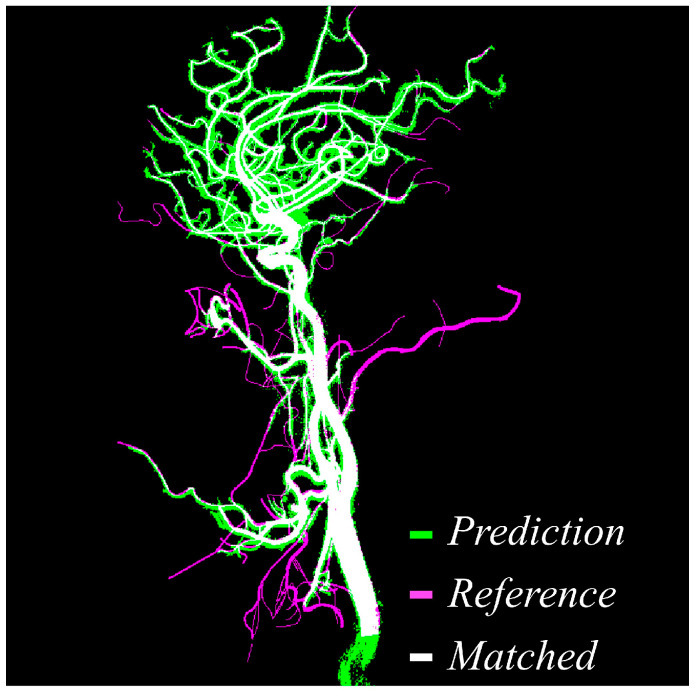
Visual comparison between predicted vessel mask and reference label. Green indicates the predicted vessels, magenta shows the reference mask, and white highlights the overlapping matched regions, demonstrating high spatial agreement between prediction and reference.

Figure 4 provides a direct visual comparison between the predicted vessel masks produced by the proposed method and the manually annotated reference labels created by expert reviewers. Within the figure, green pixels indicate areas segmented by the model, magenta pixels represent the reference ground truth, and white pixels denote the overlapping regions where prediction and reference agree. The high density of white regions across the image demonstrates the accuracy and consistency of the proposed approach. Notably, overlapping regions dominate not only the major vessels but also extend into smaller branches, which are traditionally more difficult to extract due to low contrast and noise interference. This strong correspondence confirms that the model maintains vascular continuity, successfully reduces false negatives, and delivers segmentation outputs that are closely aligned with expert interpretation.

**Figure 5:**
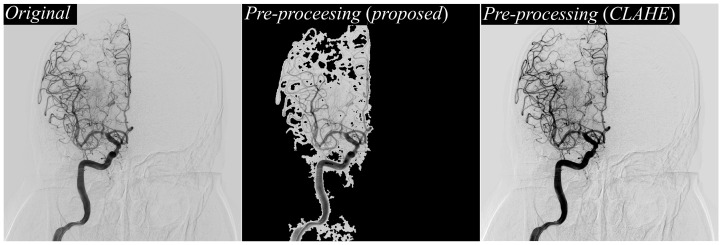
Comparison of preprocessing results on DSA images. Original DSA image (**left**), result of the proposed texture-based preprocessing method (**middle**), and result using CLAHE preprocessing (**right**), demonstrating enhanced vessel contrast and visibility differences across methods.

Figure 5 shifts focus to a side-by-side visualization of different preprocessing approaches. On the left, the original DSA image illustrates the raw input, characterized by overlapping structures, bone shadows, and background noise that often obscure vessel boundaries. In the center, the output of the proposed texture-based preprocessing method is shown, revealing more uniform and balanced enhancement of vessel regions. On the right, the CLAHE-preprocessed image is displayed for comparison. While CLAHE can enhance contrast, it tends to over-amplify bright regions, leading to uneven brightness distribution and occasional halo artifacts around bone structures. By contrast, the proposed preprocessing pipeline delivers more controlled enhancement, preserving vessel clarity while suppressing excessive amplification of non-vascular structures, which is critical for providing a clean, high-quality input to the segmentation network.

**Figure 6:**
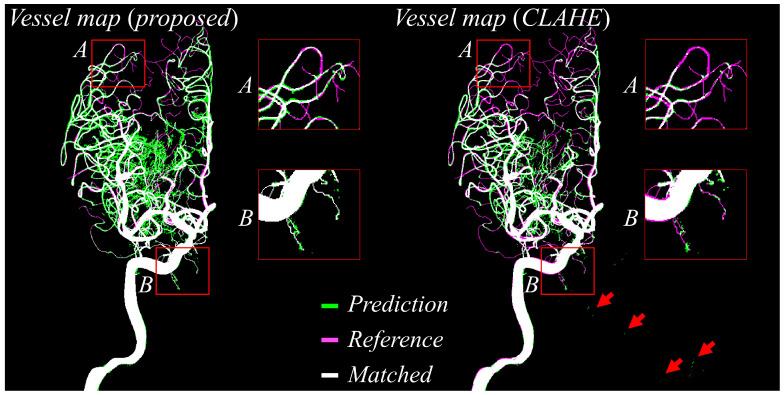
Predicted vessel maps (green) are compared against reference labels (magenta), with overlapping regions shown in white. The proposed method (**left**) shows improved alignment and fewer false positives (e.g., red arrows) than CLAHE-based preprocessing (**right**), particularly in peripheral regions (boxes A and B).

Figure 6 offers a head-to-head comparison of vessel masks predicted using the proposed preprocessing and those predicted after CLAHE preprocessing, both evaluated against the reference labels. In the visualization, green pixels represent the predicted vessels, magenta indicates the reference annotations, and white shows areas of agreement. The predictions derived from the proposed method exhibit near-complete overlap with the reference labels, as evidenced by extensive white regions covering the vessel paths. By contrast, the CLAHE-based predictions reveal several false positives (highlighted by red arrows) and fragmented vessel structures, particularly in peripheral regions labeled as boxes A and B. These fragments indicate that CLAHE introduced inconsistencies and noise, leading to misclassification of non-vascular areas and breaks in thin vessels. In contrast, the proposed approach maintains vessel connectivity across the entire vascular tree, significantly reduces noise-induced misclassifications, and produces segmentation masks that are smoother, cleaner, and more clinically interpretable.

### 3.2. Quantitative Evaluation

Table 1 summarizes the quantitative results corresponding to Figure 3 and Figure 4, reporting segmentation performance entirely in the 2D projection domain by comparing images processed without any preprocessing to those processed with the proposed texture-based preprocessing method. When the proposed approach was applied, the DICE coefficient increased from 0.65 ± 0.15 to 0.83 ± 0.20, representing a relative improvement of 27.7%. This demonstrates a substantial enhancement in pixel-level agreement between the predicted vessel masks and the manually annotated reference masks. Similarly, the IoU rose from 0.47 ± 0.20 to 0.72 ± 0.14, an approximate 53.2% relative gain, indicating that the segmented vessel regions overlapped far more closely with the ground truth. Accuracy improved from 0.81 ± 0.17 to 0.95 ± 0.15, while the F1-score climbed from 0.80 ± 0.07 to 0.93 ± 0.12, reflecting a more balanced performance between precision and recall. The sensitivity increased from 0.83 ± 0.15 to 0.88 ± 0.20, meaning that a greater proportion of true vessels were successfully detected. At the same time, specificity improved from 0.83 ± 0.15 to 0.90 ± 0.08, showing that fewer non-vascular structures were mistakenly segmented as vessels. Notably, the analysis revealed a pronounced decline in the VC metric from 115.35 ± 7.70 to 40.50 ± 5.75. This decrease of approximately 65% signifies a substantial mitigation of vascular fragmentation, thereby enhancing the structural integrity of the vasculature captured in the resulting masks.

Table 2 summarizes the quantitative results associated with Figure 5 and Figure 6, again based entirely on DSA images, and compares three preprocessing strategies: no preprocessing, CLAHE, and the proposed method. The proposed technique achieved a DICE coefficient of 0.82 ± 0.26 and an IoU of 0.75 ± 0.18. Compared to images without preprocessing (DICE 0.65 ± 0.30, IoU 0.61 ± 0.12), this represents a 26.2% increase in DICE and a 23.0% increase in IoU. Even relative to CLAHE (DICE 0.79 ± 0.41, IoU 0.70 ± 0.23), the proposed method yielded additional gains of 3.8% in DICE and 7.1% in IoU. Although the number of DSA sequences may appear limited, each sequence contains a large number of high-resolution frames, resulting in thousands of effective training samples after patch extraction. Combined with five-fold data augmentation, the overall dataset size becomes comparable to or larger than those used in previous DSA segmentation studies. Therefore, the sample size was considered adequate to train a stable nnU-Net model. A post hoc power analysis was performed based on the observed effect size for the DICE score (Cohen’s d = 0.91). Using this effect size and the number of test samples, the statistical power exceeded 0.95, indicating that the study had sufficient sensitivity to detect meaningful differences among preprocessing methods. Accuracy reached 0.97 ± 0.15 with the proposed preprocessing, compared to 0.82 ± 0.20 without preprocessing and 0.91 ± 0.25 using CLAHE, demonstrating near-perfect discrimination between vessels and background. The F1-score similarly rose to 0.96 ± 0.20, from 0.85 ± 0.10 without preprocessing and 0.91 ± 0.15 with CLAHE, confirming an improved precision–recall balance. The application of the proposed method led to a dramatic 80.2% reduction in the VC metric, from 123.27 ± 5.50 to 24.40 ± 3.38. This result signifies a profound improvement in the continuity of vascular networks compared to the unprocessed images. Compared with CLAHE, which yielded a VC of 62.73 ± 7.15, the proposed approach still reduced fragmentation by 61.1%, producing vascular masks that were far more continuous, coherent, and clinically interpretable. All quantitative results reported in Table 1 and Table 2 represent aggregate performance across the entire test set, not a selection of images. The tables summarize quantitative results over the entire test set, whereas the figures present representative qualitative examples.

Table 3 shows the runtime analysis for proposed method. Runtime evaluation showed that preprocessing required 18.2 ± 3.1 ms per frame, and nnU-Net inference required 42.5 ± 5.2 ms, resulting in a total processing time of ~60 ms per frame. The processing speed environment may be changed according to the conditions of the image photographing device actually used, and a proposed method may be implemented based on a lightweight model and performance improvement accordingly.

## 4. Discussion

The clinical results demonstrated that the proposed texture-based contrast enhancement preprocessing method significantly improved intracranial artery segmentation performance using the nnU-Net model. When compared to both the baseline condition without preprocessing and the widely used CLAHE method, the proposed approach consistently yielded superior outcomes across all key segmentation metrics, including DICE, IoU, and VC. Using the nnU-Net model, the proposed preprocessing method achieved a DICE of 0.83 ± 0.20 and an IoU of 0.72 ± 0.14. These values exceeded those obtained using CLAHE (DICE of 0.79 ± 0.41, IoU of 0.70 ± 0.23) and the baseline without preprocessing (DICE of 0.65 ± 0.15, IoU of 0.47 ± 0.20), demonstrating a clear improvement in overlap-based accuracy. One of the most notable enhancements was observed in VC, which reflects the continuity and completeness of thin and branching vascular structures. In nnU-Net, VC decreased from 115.35 ± 7.70 without preprocessing to 40.50 ± 5.75 with the proposed method, representing a 65% reduction in disconnected vessel fragments. When compared to CLAHE (VC of 62.73 ± 7.15), the proposed preprocessing still reduced fragmentation by 35.4%, confirming that the nnU-Net model preserved fine vessel branches more effectively and maintained uninterrupted vascular topology.

Although precision showed slight variation across configurations when using the proposed preprocessing, this was offset by a consistent increase in sensitivity. For example, sensitivity improved from 0.83 ± 0.15 without preprocessing to 0.88 ± 0.20 with the proposed method, indicating that nnU-Net became more adept at detecting true vascular regions, particularly smaller peripheral vessels that are often missed in conventional workflows. These findings demonstrate that the nnU-Net model, when paired with the proposed texture-based preprocessing, delivers a robust and noise-tolerant solution for intracranial artery segmentation in DSA. The substantial improvements in DICE, IoU, and VC confirm that the proposed method enhances vessel continuity and segmentation accuracy, resulting in outputs that are quantitatively superior and clinically more interpretable. In addition, the proposed preprocessing occasionally amplifies localized bright pixels that may appear as false-positive vessels, these structures are typically small, discontinuous, and morphologically distinct from true vasculature. Such artifacts can be removed through post-processing (e.g., connected-component filtering), and do not meaningfully affect the anatomical interpretability of the final segmentation.

Digital Variance Angiography (DVA) has recently gained attention as a powerful technique for enhancing vascular visibility in DSA sequences. DVA computes pixel-wise temporal variance maps across the entire image sequence, thereby amplifying contrast flow dynamics and suppressing background noise. Gyano et al. demonstrated that DVA markedly improves the contrast-to-noise ratio and vessel conspicuity in both cerebral and peripheral angiography, showing its potential as a preprocessing tool for diagnostic visualization [39]. However, DVA serves a fundamentally different purpose from the preprocessing approach proposed in our study. DVA requires access to the full temporal series and relies on inter-frame variance accumulation, whereas our framework operates at the single-frame level to generate texture-enhanced feature maps that can be directly fed into a segmentation network. Because the objective of our method is not contrast-flow visualization but structural segmentation suitable for deep learning, the computational assumptions and data requirements differ substantially. Furthermore, DVA outputs are typically continuous-valued angiographic variance maps, whereas deep learning segmentation pipelines require binarized or contrast-normalized feature representations. For these reasons, while DVA is clinically valuable for improving human interpretability, it is not directly comparable to our preprocessing pipeline, which focuses on generating segmentation-ready feature masks for nnU-Net. Nevertheless, we acknowledge that integrating DVA-derived variance maps into a deep learning workflow may offer synergetic benefits, and we intend to explore hybrid approaches in future work.

However, there remain some problems with the proposed method. First, the effectiveness of the proposed texture-based preprocessing and segmentation framework is closely tied to the quality of the DSA images provided as input. Since the method depends on texture descriptors such as local contrast, local entropy, and intensity thresholding. Its performance can be compromised by image noise, motion blur, or poor contrast between vessels and surrounding tissues. Even minor degradations, including uneven contrast agent distribution or slight motion artifacts from patient movement, may lead to inaccurate vessel enhancement and incomplete segmentation [40,41]. This reliance suggests that, while the framework performs well under optimized imaging conditions, its accuracy could fluctuate in real-world clinical settings where acquisition quality varies widely. DSA image quality itself is influenced by multiple procedural and patient-related factors, such as catheter placement, the rate of contrast injection, and a patient’s ability to remain still during imaging. Consequently, sequences captured across different hospitals may vary significantly in terms of sharpness, brightness, and artifact levels. These inconsistencies directly impact the texture feature maps that underpin the segmentation process. For instance, images with a limited dynamic range may fail to fully capture small-caliber vessels, while those with excessive noise can generate false-positive vessel signals. Such variability highlights the sensitivity of the method to acquisition conditions and underscores the importance of dataset standardization or adaptive preprocessing strategies for reliable clinical application. Mitigating this dependency on image quality is critical for transitioning the framework into a robust clinical tool. Future work should consider integrating advanced denoising and motion correction algorithms to stabilize texture information before segmentation. Furthermore, adaptive texture feature extraction could help compensate for variations in DSA acquisition and reduce performance loss in suboptimal imaging scenarios.

Second, the proposed cerebrovascular segmentation framework was developed and validated exclusively on the DIAS dataset, which provides DSA sequences focused on intracranial artery segmentation. While DIAS offers a high-quality benchmark, relying solely on a single dataset introduces concerns about data homogeneity and limited variability in imaging conditions. All sequences in DIAS were collected under similar acquisition protocols, using the same institutional practices and hardware settings. This uniformity means the nnU-Net model may have adapted specifically to DIAS characteristics rather than learning a generalizable representation of cerebrovascular anatomy across different clinical environments. As a result, the method’s performance might drop when applied to images from other hospitals, different angiography systems, or alternative procedural setups. The scarcity of sequence-based DSA data compounds this issue. Even within DIAS, the dataset contains a finite number of arterial-phase frames, limiting the range of anatomical variations, pathological presentations, and artifact profiles that the model is exposed to during training. Many rare conditions, such as subtle vascular malformations or atypical collateral vessel patterns, may be underrepresented or entirely absent. This lack of exposure can lead to biases in segmentation, where the model excels on familiar vessel appearances but struggles with unseen vascular structures or patients with atypical anatomy. Moreover, with only a small selection of sequences available, the model risks overfitting to dataset-specific textures and noise patterns, further reducing its generalizability [42,43]. Overcoming this limitation will require expanding beyond DIAS to incorporate multi-institutional, multi-device DSA datasets that capture broader variability in anatomy, acquisition protocols, and patient demographics. Synthetic data augmentation strategies, such as generating realistic DSA sequences through simulation or applying domain randomization techniques, could help diversify the training set when real data are limited. Additionally, future work should evaluate the proposed method on external test sets to verify its robustness and adapt the preprocessing pipeline to handle the variability introduced by different clinical sources. Addressing these gaps will be crucial for transitioning the framework from a proof-of-concept research tool into a generalizable and clinically reliable solution. Because the DIAS dataset originates from a single institution, performance may vary across scanners, acquisition protocols, or patient motion conditions. Future work will evaluate the method using multi-center datasets and prospective clinical data to assess robustness under real-world imaging variability. In addition, this study did not separately evaluate the segmentation performance between vascular-lightweight categories (e.g., small, medium, and large blood vessels). This stratification analysis requires clinically validated definition of arterial diameter range and coordinated annotation with a neurointerventionist and is very important in terms of adding an evaluation shortfall in the DIAS dataset. Future studies will perform clinically induced vascular size classification and statistical analysis to better assess the sensitivity of the model to vascular branching.

Finally, it is a problem of sensitivity of pretreatment parameters and computational efficiency in clinical applications. The proposed cerebrovascular segmentation framework relies on texture-based preprocessing steps to enhance vessel visibility before nnU-Net segmentation. However, these processes introduce an inherent sensitivity to parameter settings including window size, entropy thresholds, and brightness cut-offs. Even minor adjustments to these parameters can substantially alter the resulting feature maps, determining which structures are enhanced or suppressed. As a result, segmentation outcomes may vary across datasets, operators, or imaging conditions. Parameters optimized for the DIAS dataset, which was acquired under consistent protocols, may not transfer effectively to DSA data from other hospitals or imaging systems. Overly aggressive preprocessing could eliminate faint vessels, while conservative settings might allow excessive noise or bone edges to obscure segmentation, limiting the method’s generalizability and requiring manual parameter tuning for each new dataset. In addition, its computational efficiency remains a critical barrier to real-time clinical use while the proposed method delivers strong segmentation performance. The texture-based preprocessing pipeline and nnU-Net inference create significant processing overhead, particularly when applied to long DSA sequences. This latency is problematic in time-sensitive neurovascular procedures, such as stroke thrombectomy, where clinicians need near-instantaneous vessel visualization. Previous studies have explored similar challenges: Meng et al. introduced a phase analysis approach to restore vessel continuity in limited 15 FPS sequences [44], while Haouchine et al. developed patch-based interpolation techniques to predict high-frame-rate (30 FPS) sequences in low-dose DSA [45]. These works show that leveraging temporal information and efficient data-driven reconstruction can address computational bottlenecks in demanding angiographic applications. To address these limitations, future work should focus on both adaptive preprocessing and computational optimization. Adaptive strategies that dynamically adjust parameters based on local image quality or learn optimal settings directly within the neural network could minimize manual tuning, improving robustness across diverse clinical conditions. At the same time, enhancing computational efficiency through GPU-based acceleration, consolidating preprocessing into a single learned module, and employing lightweight nnU-Net–derived architectures would significantly reduce processing time. Through continuous R&D, there are plans to advance the proposed algorithm considering this point in future research.

## 5. Conclusions

This study proposed a texture-based contrast enhancement preprocessing framework integrated with the nnU-Net model to improve intracranial artery segmentation in time-sequential DSA images. By extracting and fusing local contrast, local entropy, and brightness threshold feature maps, the method provided enhanced vessel-focused inputs that significantly improved segmentation performance across all evaluated metrics. Compared to both the baseline without preprocessing and the widely used CLAHE method, the proposed approach demonstrated marked gains in DICE, IoU, and vessel connectivity, with VC decreasing by over 65% compared to unprocessed images, indicating a substantial improvement in vascular continuity and topological integrity. Despite these advances, limitations remain, including dependence on DSA image quality, reliance on the single-center dataset, and sensitivity to preprocessing parameters, as well as computational demands that currently hinder real-time application. Future research should address these issues by incorporating multi-institutional datasets, adaptive parameter tuning, and computational optimizations to enable clinical deployment. In conclusion, the proposed framework demonstrates that combining texture-based preprocessing with deep learning can deliver robust, noise-tolerant, and clinically interpretable vessel segmentation in DSA. With further refinement, this approach has strong potential to support automated cerebrovascular assessment in routine clinical workflows, enhancing diagnostic accuracy and efficiency in neurovascular care.

## Figures and Tables

**Table 1:** Quantitative performance comparison of cerebral vessel segmentation in the projection domain according to different preprocessing methods including the proposed texture-based approach.

Pre-Processing Method	Parameter	Mean	Std.	95% Cl
None	DICE	0.65	0.15	[0.61 0.69]
	IoU	0.47	0.20	[0.42 0.52]
	Accuracy	0.81	0.17	[0.77 0.85]
	Sensitivity	0.83	0.15	[0.79 0.87]
	Specificity	0.83	0.15	[0.75 0.85]
	Precision	0.80	0.20	[0.75 0.85]
	F1-score	0.80	0.07	[0.77 0.85]
	Vessel connectivity	115.35	7.70	[113.40 117.30]
Proposed	DICE	0.83	0.20	[0.78 0.88]
	IoU	0.72	0.14	[0.68 0.76]
	Accuracy	0.95	0.15	[0.83 0.97]
	Sensitivity	0.88	0.20	[0.83 0.93]
	Specificity	0.90	0.08	[0.86 0.94]
	Precision	0.88	0.15	[0.84 0.92]
	F1-score	0.93	0.12	[0.90 0.96]
	Vessel connectivity	40.50	5.75	[39.05 41.95]

**Table 2:** Performance evaluation of cerebral vessel segmentation using the pre-processing method including the none, the proposed, and the CLAHE.

Pre-Processing Method	Parameter	Mean	Std.	95% Cl
None	DICE	0.65	0.30	[0.57 0.73]
	IoU	0.61	0.12	[0.54 0.68]
	Accuracy	0.82	0.20	[0.76 0.84]
	Sensitivity	0.83	0.15	[0.80 0.86]
	Specificity	0.82	0.15	[0.82 0.88]
	Precision	0.85	0.10	[0.82 0.88]
	F1-score	0.85	0.10	[0.82 0.88]
	Vessel connectivity	123.27	5.50	[121.88 124.66]
Proposed	DICE	0.82	0.26	[0.75 0.89]
	IoU	0.75	0.18	[0.69 0.81]
	Accuracy	0.97	0.15	[0.95 0.99]
	Sensitivity	0.95	0.18	[0.91 0.97]
	Specificity	0.95	0.18	[0.90 0.99]
	Precision	0.96	0.20	[0.94 0.98]
	F1-score	0.96	0.20	[0.94 0.98]
	Vessel connectivity	24.40	3.38	[23.64 25.16]
CLAHE	DICE	0.79	0.41	[0.69 0.90]
	IoU	0.70	0.23	[0.64 0.76]
	Accuracy	0.91	0.25	[0.89 0.93]
	Sensitivity	0.85	0.12	[0.82 0.93]
	Specificity	0.85	0.12	[0.82 0.94]
	Precision	0.91	0.15	[0.89 0.95]
	F1-score	0.91	0.15	[0.89 0.93]
	Vessel connectivity	62.73	7.15	[60.92 64.54]

**Table 3:** Performance evaluation of processing time to perform the proposed framework.

	Pre-Processing	nnU-Net
Processing time (ms/frame)	18.2 ± 3.1	42.5 ± 5.2

## Data Availability

The Raw data supporting the conclusions of this study will be available upon request by authors.
